# Differential response to dexamethasone on the TXB_2_ release in guinea-pig alveolar macrophages induced by zymosan and cytokines

**DOI:** 10.1080/09629359791523

**Published:** 1997-12

**Authors:** M. E. Salgueiro, M. Conde, A. J. Seco, N. Méndez, G. Manso

**Affiliations:** 1 Departamento de Medicina Farmacología Facultad de Medicina Julián Clavería s/n Oviedo 33006 Spain

## Abstract

Glucocorticosteroids reduce the production of inflammatory mediators but this effect may depend on the stimulus. We have compared the time course of the effect of dexamethasone on the thromboxane B_2_ (TXB_2_) release induced by cytokine stimulation and zymosan in guinea-pig alveolar macrophages. Interleukin-1β (IL-1β), tumour necrosis factor-α (TNF-α) and opsonized zymosan (OZ), all stimulate TXB_2_ release. High concentrations of dexamethasone (1–10 μM) inhibit the TXB_2_ production induced by both cytokines and OZ, but the time course of this response is different. Four hours of incubation with dexamethasone reduce the basal TXB_2_ release and that induced by IL-1β and TNF-α, but do not modify the TXB_2_ release induced by OZ. However, this stimulus was reduced after 24 h incubation. Our results suggest that the antiinflammatory activity of glucocorticosteroids shows some dependence on stimulus and, therefore, may have more than one mechanism involved.


Mediators of Inflammation, 6, 375–380 (1997)

### Differential response to dexamethasone on the $TXB_2$ release in guinea-pig alveolar macrophages induced by zymosan and cytokines

M. E. Salgueiro, M. Conde, A. J. Seco, N. Méndez and G. Manso[CA]

Departamento de Medicina, Farmacología, Facultad de Medicina, Julián Clavería s/n, 33006 Oviedo, Spain

[CA]Corresponding Author
Tel: (+34) 85 103546
Fax: (+34) 85 232255

**Glucocorticosteroids reduce the production of inflammatory mediators but this effect may depend on the stimulus. We have compared the time course of the effect of dexamethasone on the thromboxane $B_2$ ($TXB_2$) release induced by cytokine stimulation and zymosan in guinea-pig alveolar macrophages. Interleukin-1β (IL-1β), tumour necrosis factor-α (TNF-α) and opsonized zymosan (OZ), all stimulate $TXB_2$ release. High concentrations of dexamethasone (1–10 μM) inhibit the $TXB_2$ production induced by both cytokines and OZ, but the time course of this response is different. Four hours of incubation with dexamethasone reduce the basal $TXB_2$ release and that induced by IL-1β and TNF-α, but do not modify the $TXB_2$ release induced by OZ. However, this stimulus was reduced after 24 h incubation. Our results suggest that the anti-inflammatory activity of glucocorticosteroids shows some dependence on stimulus and, therefore, may have more than one mechanism involved.**

**Key words:** Dexamethasone, Interleukin-1β, Tumour necrosis factor-α, Opsonized zymosan, Thromboxane $B_2$, Macrophages

### Introduction

Alveolar macrophages are distributed widely in the airways and lung parenchyma. They release a range of mediators including those derived from arachidonic acid such as thromboxane $A_2$ ($TXA_2$), which is rapidly converted to its stable metabolite thromboxane $B_2$ ($TXB_2$), and proinflammatory cytokines such as interleukin 1β (IL-1β) and tumour necrosis factor α (TNF-α).[1] These cytokines act on other inflammatory cells and also exert an autocrine control of macrophages.[2] The preincubation with IL-1β or TNF-α induces the production of metabolites of arachidonic acid in peritoneal macrophages,[3] monocytes,[4] neutrophils,[5] endothelial cells[6] and synovial cells.[7] The action of these cytokines is probably due to an increase in the activity of enzymes involved in the activation of membrane phospholipids. In some inflammatory cells, IL-1β and TNF-α induce the synthesis of the cytosolic and secreted phospholipase $A_2$ group II ($cPLA_2$ and $sPLA_2$ group II)[8,9] and the inducible form of cyclooxygenase (COX-2).[10–12] It has recently been described that some of the pro-inflammatory effects induced by IL-1β and TNF-α are mediated by the activation of the transcription factors, activating protein-1 (AP-1) and nuclear factor kappa B (NFκB).[13] In some inflammatory cells, phagocytosis of opsonized zymosan (OZ) stimulates the release of eicosanoids and this effect seems to be independent of the production of specific forms of $PLA_2$ and COX.

Glucocorticosteroids are an effective treatment for controlling inflammatory diseases. Their biological effects are produced in part by the alteration of gene expression in their target cells. By binding to the DNA, glucocorticosteroids modulate the synthesis of proteins regulating the production and the effects of inflammatory mediators.[14] One system inhibited by glucocorticosteroids seems to be the reduction of eicosanoid release. Inhibition of prostaglandin synthesis by *in vitro* glucocorticosteroids in monocytes,[15] macrophages[16,17] and endothelial cells[18] has been reported. To explain this inhibitory effect, two different mechanisms have been proposed: (1) the induction of the synthesis of lipocortins, which act as antagonists of $PLA_2$[19,20] and (2) the direct inhibition of the synthesis and release of $PLA_2$[21] and COX-2[22,23] induced by cytokines. In some animal models, the production of lipocortins has also been proposed as the mechanism by which glucocorticoids inhibit the eicosanoid release induced by OZ.[19] However, whether this mechanism of glucocorticosteroid action is the same for all systems has not been investigated.

© 1997 Rapid Science Publishers 

The aim of this study was to compare the time course and the concentration response to dexamethasone on the $TXB_2$ release induced by OZ and the proinflammatory cytokines IL-1β and TNF-α.

### Materials and Methods

### Materials

Zymosan A, dexamethasone, $TXB_2$, antiserum to $TXB_2$, IL-1β, TNF-α, trizma base, albumin bovine (BSA), dextran and activated charcoal were obtained from Sigma Chemical Co. (St Louis, MO); Dulbecco's Modified Eagle's Medium (DMEM) and phosphate-buffered saline (PBS) from GIBCO (Grand Island, NY); May-Grünwald-Giemsa from Merck (Germany); $[^3H]TXB_2$ (120 Ci/mmol) from Amersham International (UK); scintillation cocktail from (Scharlau, Spain) and 24-well culture plates from Corning (NY). The biological activities of IL-1β and TNF-α were $5 \times 10^7$ U/mg and $2 \times 10^7$ U/mg, respectively. Zymosan was opsonized by incubation with pooled guinea pig serum for 30 min at 37°C, and used after centrifugation at 1800 rpm room temperature for 3 min and washing in PBS four times.

### Methods

*Alveolar macrophage isolation and culture*
Male Dunkin-Hartley guinea pigs weighing about 400 g were used. The guinea pigs were anaesthetized by intraperitoneal injection of sodium thiopental (50 mg/kg) (Braun Medical SA, Spain). The trachea was cannulated and the heart and lungs were removed *en bloc*. The lungs were lavaged in 9 ml aliquots, for a total volume of 36 ml sterile PBS and gently massaged with each infusate in order to maximize the cell yield. The cell suspension was washed twice by centrifugation at 1000 rpm at room temperature for 10 min, and further dissolution in 20 ml of sterile PBS. The total cell count was determined on a grid haemocytometer. A typical lavage yielded $0{\cdot}8-1{\cdot}8 \times 10^7$ cells comprised of 90–95% macrophages as confirmed by the May-Grünwald-Giemsa dye. Afterwards, alveolar macrophages were resuspended in DMEM at a density of $3 \times 10^5$ cells/ml, placed in the 24-well plates (1 ml/well) and incubated at 37°C, in a humidified 95% air/5% $CO_2$ atmosphere, for 1 h, to allow adherence. Then, the cells were washed three times with fresh DMEM and the drug exposure was performed. Guinea-pig alveolar macrophages were exposed to OZ (10–1000 μg/ml PBS), IL-1β (0.1–10 ng/ml PBS 0.1% BSA) or TNF-α (0.1–10 ng/ml PBS 0.1% BSA) for 1 h or 4 h. After this time, the supernatant was harvested and added to 0.5 M TRIS HCl pH 7.4, in a ratio 9:1, and stored at −20°C for the later analysis of $TXB_2$. Each experiment was made in duplicate. The effects of all solvents used were tested. None affected the cellular response.

To study the effect of glucocorticosteroids on the $TXB_2$ release induced by OZ, macrophages were incubated during 4 h or 24 h in the presence or absence of dexamethasone (0.1–100 μM PBS 0.01% ethanol). Following this incubation, the cells were washed twice with fresh DMEM and challenged to OZ (1 mg/ml PBS) for 1 h. In further experiments, macrophages were incubated with several concentrations of OZ (10–1000 μg/ml) in the presence or absence of a fixed high concentration of dexamethasone (10 μM) for 4 h.

To analyse the effect of glucorticosteroids on the $TXB_2$ release induced by cytokines, macrophages were incubated during 4 h with a fixed concentration of IL-1β (1 ng/ml) or TNF-α (3 ng/ml) and several concentrations of dexamethasone (0.1–100 μM) for 4 h and, in other experiments, with several concentrations of IL-1β (0.1–10 ng/ml) or TNF-α (0.1–10 ng/ml) in the presence or absence of dexamethasone (10 μM) during 4 h. At the end of the incubation time, the supernatant was harvested as described above.

*Radioimmunoassay to $TXB_2$*
$TXB_2$ in macrophage supernatants was determined by radioimmunoassay (RIA) as previously described by Fuller *et al.*[16] Briefly, 50 μl of standard $TXB_2$ (or unknown samples) and 50 μl of $[^3H]TXB_2$ were incubated with 50 μl of diluted antiserum to $TXB_2$ overnight at 4°C. Bound radioactivity was separated from free radioactivity by addition of 1 ml of dextran coated charcoal and centrifugation at 2800 rpm 4°C 20 min. The supernatants were transferred to vials and diluted in 1 ml of scintillation cocktail. A calibration curve was established with standard $TXB_2$ in a range from 1 to 250 pg/0.1 ml. Assays were run in duplicate. The level of sensitivity was 4 pg/0.1 ml. The anti-$TXB_2$ serum cross-reacted 0.5% $PGF_{2\alpha}$ and was less than 0.1% $PGF_{1\alpha}$, $PGD_2$, $PGE_1$, 6-keto $PGF_{1\alpha}$. Results obtained in the RIAs are expressed as picograms of $TXB_2$ released per $10^5$ macrophages or as the percentage of the maximum $TXB_2$ released in each experiment.

### Statistical analysis

Data are presented as the mean ± SEM. Statistical analysis was performed by unpaired Student's *t*-test (for two groups) or ANOVA (for multiple comparisons). Differences between values were considered significant if $P < 0{\cdot}05$.

### Results

### $TXB_2$ production induced by OZ, IL-1β and TNF-α

The concentration and time-dependent effect of OZ is shown in Fig. 1. Guinea-pig alveolar macrophages incubated with OZ (0.01–1 mg/ml) showed an enhanced production of $TXB_2$. This increase was significant after both 1 and 4 h of incubation, and the amount of unstimulated and stimulated $TXB_2$ was greater after 4 h than after 1 h ($P < 0{\cdot}01$). In both cases, the maximum increase obtained in this study was with 1 mg/ml of OZ. Cells exposed to this concentration, for 4 h, released 258·6 ± 15·0 pg of $TXB_2/10^5$ cells.

The effect of the incubation with IL-1β (0.1–10 ng/ml) and TNF-α (0.1–10 ng/ml), for 4 h, on the production of $TXB_2$ is shown in Fig. 2. There was a small, but significant ($P < 0{\cdot}05$), concentration-dependent increase on the mediator release after 4 h, but not after 1 h (Table 1) of incubation with both cytokines. IL-1β and TNF-α induced a similar level of stimulation. The maximum increase was also similar: 109·3 ± 19·0 pg/$10^5$ cells to 3 ng/ml of IL-1β, and 118·8 ± 14·3 pg/$10^5$ cells to 3 ng/ml of TNF-α. This value was lower than that induced by OZ.

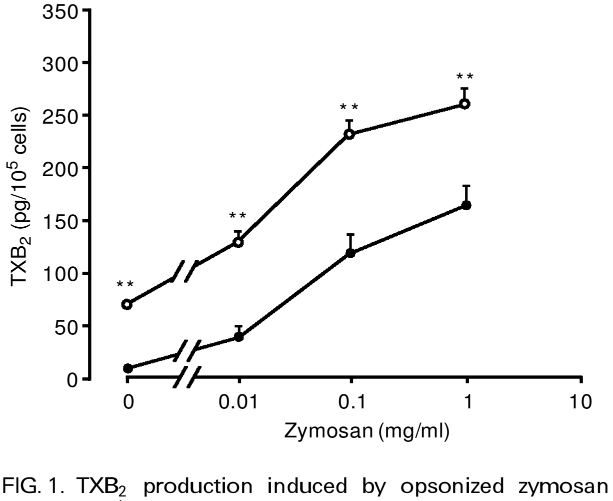

FIG. 1. $TXB_2$ production induced by opsonized zymosan (0.01–1 mg/ml), for 1 h (filled circles) or 4 h (open circles), from guinea-pig alveolar macrophages. $^{**}P < 0.01$, compared with the results obtained after 1 h of incubation. $n \geq 6$.

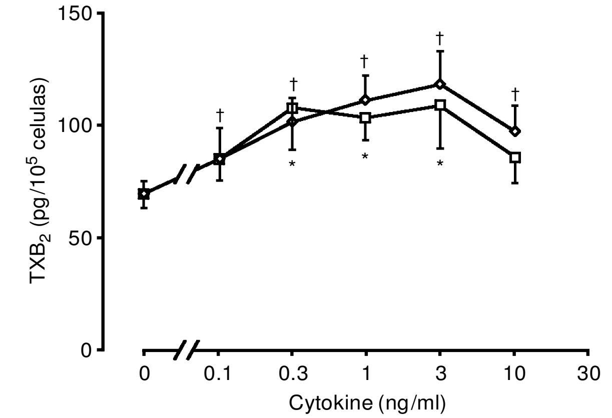

FIG. 2. $TXB_2$ production induced by IL-1β (0.1–10 ng/ml) (open squares) or TNF-α (0.1–10 ng/ml) (open diamonds), for 4 h, from guinea-pig alveolar macrophages. Asterisks indicate the statistical significance to IL-1β and daggers to TNF-α. $^{*}P < 0.05$, $^{\dagger}P < 0.05$, compared with the control (0). $n \geq 6$.

**Table 1**. The effect of the incubation with cytokines for 1 h

| Addition | $TXB_2$ (pg/$10^5$ cells) |
|---|---|
| Control | 9.2 ± 2.5 |
| IL-1β (ng/ml) | |
| 0.3 | 9.7 ± 2.7 |
| 1 | 9.0 ± 2.3 |
| 3 | 10.0 ± 2.5 |
| 10 | 16.0 ± 5.8 |
| TNF-α (ng/ml) | |
| 3 | 9.7 ± 3.0 |
| 10 | 10.4 ± 1.7 |

None of the values is significant compared with control. $n \geq 5$.

### Effect of dexamethasone on the zymosan-induced release

In Fig. 3, the incubation with dexamethasone (10 μM) for 4 h did not alter the concentration response to zymosan (0.01–1 mg/ml), although it significantly ($P < 0{\cdot}05$) reduced the unstimulated release of $TXB_2$. Fig. 4 shows that the preincubation for 24 h ($P < 0{\cdot}01$), but not 4 h, with dexamethasone (0.1–100 μM) inhibited $TXB_2$ release induced by OZ (1 mg/ml).

### Effect of dexamethasone on the IL-1β or TNF-α release

The effect of the joint incubation with dexamethasone and IL-1β or TNF-α, for 4 h, is shown in Fig. 5. When dexamethasone (0.1–100 μM) was added with a fixed concentration of IL-1β (1 ng/ml) or TNF-α (3 ng/ml), concentration dependent inhibition of $TXB_2$ release was observed, and this was significant ($P < 0{\cdot}05$) up to 1 μM to IL-1β and ($P < 0{\cdot}01$) up to 10 μM to TNF-α (Fig. 5A). The incubation with a fixed high concentration of dexamethasone (10 μM)

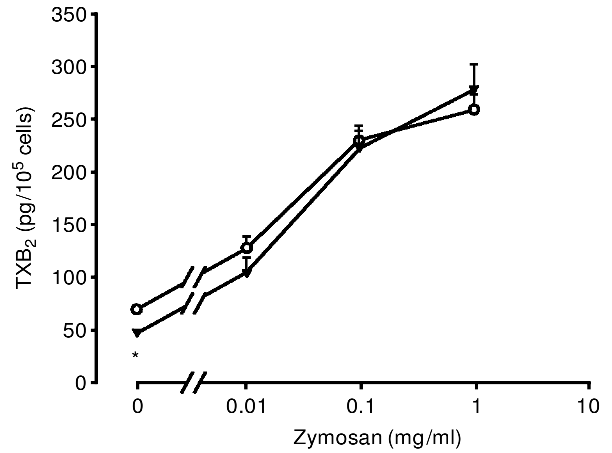

FIG. 3. The effect of the incubation with opsonized zymosan (0.01–1 mg/ml) in the absence (open circles) or presence (filled circles) of dexamethasone (10 μM), for 4 h, on the $TXB_2$ release. $^*P < 0.05$, compared with the control. $n \geqslant 6$.

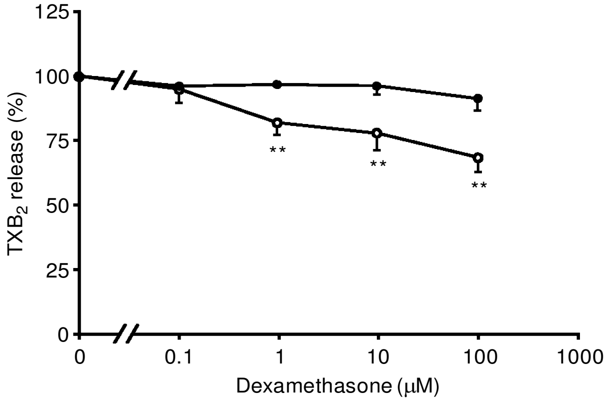

FIG. 4. The effect of the preincubation with dexamethasone (0.1–100 μM), for 4 h (filled circles) or 24 h (open circles), on the $TXB_2$ release induced by the further incubation, for 1 h, with opsonized zymosan (1 mg/ml). $^{**}P < 0.01$, compared with the control (100% = release induced by 1 mg/ml of opsonized zymosan). $n \geqslant 6$.

significantly ($P < 0{\cdot}05$) inhibited both unstimulated $TXB_2$ and the concentration response to IL-1β (0.1–10 ng/ml) or TNF-α (0.1–10 ng/ml) (Fig. 5B).

### Discussion

Incubation of guinea-pig alveolar macrophages with IL-1β, TNF-α and OZ increase the release of $TXB_2$. However, this release has a different time course and magnitude of effects. OZ caused a more rapid and greater release of $TXB_2$ either of the cytokines. A difference was also found in the effects of dexamethasone on the release induced by OZ and cytokines. Incubation with dexamethasone, for 4 h, reduced the unstimulated $TXB_2$ release in all studies and the cytokines induced increase in release. No effect after 4 h incubation was obtained on the increase induced by OZ even at lower concentrations. However, after 24 h incubation the effect of OZ was reduced in a concentration dependent fashion by dexamethasone.

The effects of the three stimuli used here have been previously studied in other inflammatory cells. Opsonized zymosan induces the eicosanoid release from rat peritoneal leukocytes,[17] human monocytes[15] and human macrophages[16] after 1 h of incubation and these results are consistent with our observations on guinea-pig alveolar macrophages. The elimination of pathogens from human airways is dependent to a great extent on the capacity of resident macrophages to phagocytose, kill and finally digest bacteria. Phagocytosis of OZ rapidly activates macrophages to release numerous lysosomal hydrolases, oxygen radicals and

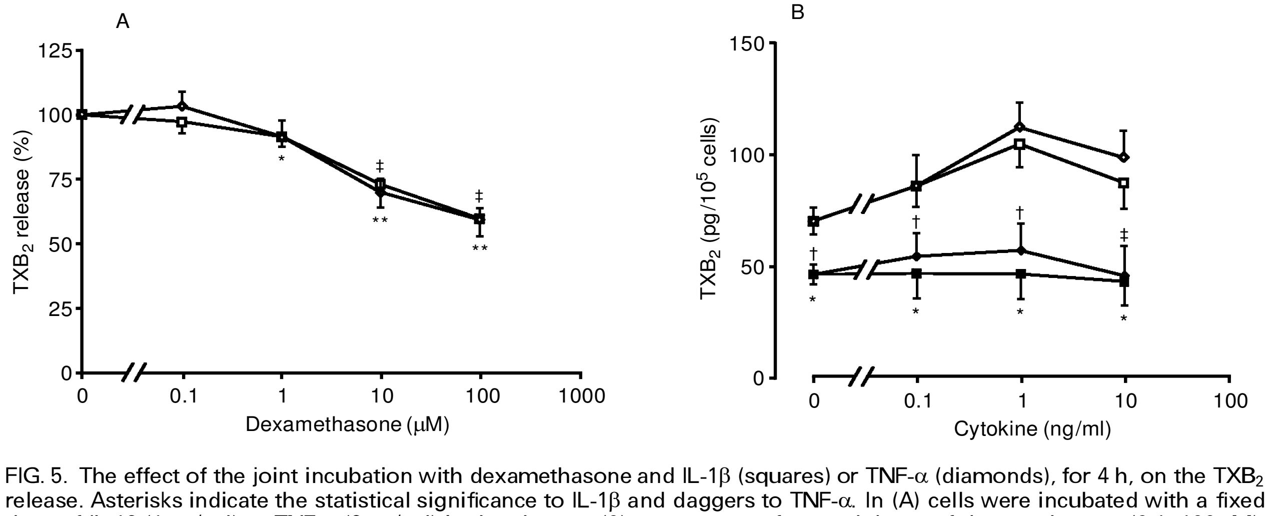

FIG. 5. The effect of the joint incubation with dexamethasone and IL-1β (squares) or TNF-α (diamonds), for 4 h, on the $TXB_2$ release. Asterisks indicate the statistical significance to IL-1β and daggers to TNF-α. In (A) cells were incubated with a fixed dose of IL-1β (1 ng/ml) or TNF-α (3 ng/ml) in the absence (0) or presence of several doses of dexamethasone (0.1–100 μM). $^*P < 0.05$, $^{**}P < 0.01$, $^‡P < 0.01$, compared with the control (100%). $n \geqslant 6$. In (B) cells were incubated with several doses of IL-1β (0.1–10 ng/ml) or TNF-α (0.1–10 ng/ml) in the absence (open symbols) or presence (filled symbols) of a fixed dose of dexamethasone (10 μM). $^*P < 0.05$, $^{**}P < 0.01$, $^†P < 0.05$, $^‡P < 0.01$, compared with the results obtained in the absence of dexamethasone. $n \geqslant 6$.

products of cycloxygenase and lipoxygenase reproducing partially the response to infective stimuli.[24] In our experiments, IL-1β and TNF-α also activate the $TXB_2$ release from guinea-pig alveolar macrophages but their effect is less pronounced and delayed compared with the one obtained with OZ. The results are consistent with studies in other inflammatory cells, where an increase on the eicosanoid release induced by IL-1β and TNF-α has been shown.[3,5–7] This effect requires long periods of time to appear, beginning after 4 h and reaching the maximum response after 12–24 h, depending on the type of cell, thus suggesting that it could be mediated by an increase of the synthesis of proteins. The incubation with cycloheximide and actinomycin D abolish the release of $PGE_2$ induced by IL-1β from human fibroblasts.[10] Many cytokines, including IL-1β and TNF-α activate the transcription factors AP-1 and NFκB which regulate the gene transcription of many target genes, including enzymes, receptors and cytokines themselves[13] and the incubation with cytokines induces the production of some enzymes involved in the phospholipid breakdown. The increase of the production of $cPLA_2$,[9] $sPLA_2$ group II[8,21] and COX-2[11,12] induced by IL-1β and TNF-α has been described in other inflammatory cells. These enzymes could be responsible for the increase of $TXB_2$ induced by cytokines observed in our experiments. However, whether or not the production of $cPLA_2$, $sPLA_2$ group II and COX-2, is mediated by the activation of AP-1 or NFκB has not yet been established.

In guinea-pig alveolar macrophages, dexamethasone inhibits the $TXB_2$ release induced by OZ or cytokines, but the time course of this response is different depending on the stimulus. To explain the reduction of mediators derived from arachidonic acid induced by glucocorticosteroids, mechanisms such as gene binding and increase of lipocortins have been proposed. Although lipocortin-1 inhibits the eicosanoid release from rat and human macrophages,[20] some authors have been unable to demonstrate induction of lipocortins to explain the reduction of the eicosanoids induced by glucocorticosteroids.[18,25] In PMA-differentiated U937 cells the incubation with dexamethasone for 16 h, but not 4 h, caused an increase on the mRNA level of lipocortin-1 and 2.[26] This time course is similar to the one found in our experiments on the $TXB_2$ release induced by OZ and this could be the mechanism involved in the delayed effect of dexamethasone. However, 4 h of incubation with dexamethasone inhibits the $TXB_2$ release induced by IL-1β or TNF-α and the mechanism involved in this effect could be different from that observed in the release induced by OZ. In several experimental models, glucocorticosteroids and cytokines have shown opposite effects on the synthesis of proteins.[14] Dexamethasone inhibits the AP-1 and NFκB DNA binding induced by IL-1β or TNF-α in human peripheral blood mononuclear cells[27] and combined treatment with dexamethasone and TNF-α prevent the increase in both AP-1 and NFκB binding due to TNF-α in human lung.[28] Glucocorticosteroids also prevent the activation of specific enzymes involved in the eicosanoid synthesis induced by cytokines. Dexamethasone blocks the increase in $cPLA_2$,[9] $sPLA_2$ group II[21] and COX-2[23,29] mediated by cytokines. A specific interaction between cytokines and glucocorticosteroids could explain the rapid inhibitory effect of dexamethasone on the $TXB_2$ release induced by IL-1β or TNF-α found in our experiments. However, high concentrations of dexamethasone also inhibit the basal $TXB_2$ release after 4 h of incubation. This effect could be related to a more rapid effect of dexamethasone at lower levels of stimulation or to the increase of $sPLA_2$ group II described after the culture of macrophages.[30]

In conclusion, we have found a different time course on the effect of dexamethasone depending on the stimulus. Basal mediator release and that induced by cytokines are rapidly prevented by dexamethasone and this effect could be mediated by the inhibition of transcription factors or enzymes activated during the inflammation. However, the mediator release induced by phagocytic stimuli requires longer incubation for reduction by dexamethasone and this effect could be due to a longer mechanism, such as gene binding or release of lipocortins. These results support the observation that glucocorticosteroids may not have an exclusive mechanism to reduce the release of mediators derived from arachidonic acid.

### References

1. Lohmann-Mathes ML, Steinmüller C, Franke-Ullman G. Pulmonary macrophages. *Eur J Respir Dis* 1994; **7**: 1678–1684.
2. Elias JA, Zitnik RJ. Cytokine-cytokine interactions in the context of cytokine networking. *Am J Respir Cell Mol Biol* 1992; **7**: 365–367.
3. Bachwich PR, Chensue SW, Larrick JW, Kunkel SL. Tumor necrosis factor stimulates interleukin-1 and prostaglandin $E_2$ production in resting macrophages. *Biochem Biophys Res Commun* 1986; **136**: 94–101.
4. Conti P, Panara MR, Barbacane RC, et al. Blocking the interleukin-1 receptor inhibits leukotriene $B_4$ and prostaglandin $E_2$ generation in human monocytes cultures. *Cell Immunol* 1992; **145**: 199–209.
5. Akama H, Ichikawa Y, Matsushita Y, Shinoza T, Homma M. Mononuclear cells enhance prostaglandin $E_2$ production of polymorphonuclear leukocytes via tumor necrosis factor α. *Biochem Biophys Res Commun* 1990; **168**: 857–862.
6. Albrightson CR, Baenziger NL, Needleman P. Exaggerated human vascular cell prostaglandin biosynthesis mediated by monocytes: role of monokines and interleukin-1. *J Immunol* 1985; **135**: 1872–1877.

7. O'Neill LA, Lewis GP. Interleukin 1 potentiates bradykinin- and TNF alpha-induced $PGE_2$ release. *Eur J Pharmacol* 1989; **166**: 131–137.
8. Vadas P, Pruzanski W, Stefanski E, *et al.* Extracellular phospholipase A2 secretion is a common effector pathway of interleukin-1 and tumor necrosis factor action. *Immunol Lett* 1991; **28**: 187–194.
9. Lih-Ling L, Lin AJ, DeWitt DL. Interleukin-1$\alpha$ induces the accumulation of cytosolic phospholipase $A_2$ and the release of prostaglandin $E_2$ in human fibroblasts. *J Biol Chem* 1992; **267**: 23451–23454.
10. Raz A, Wyche A, Siegel N, Needleman P. Regulation of fibroblast cyclooxygenase synthesis by interleukin-1. *J Biol Chem* 1988; **263**: 3022–3028.
11. Maier JAM, Hla T, Maciag T. Cyclooxygenase is an immediate-early gene induced by interleukin-1 in human endothelial cells. *J Biol Chem* 1990; **265**: 10805–10808.
12. Lee SH, Soyoola E, Chanmugam P, *et al.* Selective expresssion of mitogen-inducible cyclooxygenase in macrophages stimulated with lipopolysaccharide. *J Biol Chem* 1992; **267**: 25934–25938.
13. Barnes PJ. Cytokines as mediators of chronic asthma. *Am J Respir Crit Care Med* 1994; **150**: S42–S49.
14. Barnes PJ, Adcock I. The anti-inflammatory actions of steroids: molecular mechanisms. *Trends Pharmacol Sci* 1993; **14**: 436–441.
15. Manso G, Baker AJ, Taylor IK, Fuller RW. In vivo and in vitro effects of glucocorticosteroids on arachidonic acid metabolism and monocyte function in nonasthmatic patients. *Eur J Respir Dis* 1992; **5**: 712–716.
16. Fuller RW, Kelsey CR, Cole PJ, Dollery CT, MacDermot J. Dexamethasone inhibits the production of thromboxane $B_2$ and leukotriene $B_4$ by human alveolar peritoneal macrophages in culture. *Clin Sci* 1984; **67**: 653–656.
17. Parente L, Flower RJ. Hydrocortisone and macrocortin inhibit the zymosan-induced release of lyso-PAF from rat peritoneal leucocytes. *Life Sci* 1985; **36**: 1225–1231.
18. Hullin F, Raynal P, Ragab-Thomas JMF, Fauvel J, Chap H. Effect of dexamethasone on prostaglandin synthesis and on lipocortin status in human endothelial cells. *J Biol Chem* 1989; **264**: 3506–3513.
19. Peers SH, Flower RJ. The role of lipocortin in corticosteroid actions. *Am Rev Respir Dis* 1990; **141**: S18–S21.
20. Flower RJ, Rothwell NJ. Lipocortin-1: cellular mechanisms and clinical relevance. *Trends Pharmacol Sci* 1994; **15**: 71–76.
21. Schalkwijk C, Vervoordeldonk M, Pfeilschifter J, Märki F, van den Bosch H. Cytokine- and forskolin-induced synthesis of group II phospholipase $A_2$ and prostaglandin $E_2$ in rat mesangial cells is prevented by dexamethasone. *Biochem Biophys Res Commun* 1991; **180**: 46–52.
22. O'Banion MK, Winn VD, Young DA. cDNA cloning and functional activity of a glucocorticoid-regulated inflammatory cyclooxygenase. *Proc Natl Acad Sci USA* 1992; **89**: 4888–4892.
23. Mitchell JA, Belvisi MG, Akarasereenont P, *et al.* Induction of cyclooxygenase-2 by cytokines in human pulmonary epithelial cells: regulation by dexamethasone. *Br J Pharmacol* 1994; **113**: 1008–1014.
24. MacDermot J, Fuller RW. Macrophages. In: Barnes PJ, Rodger IW, Thomson NC, eds. *Asthma. Basic Mechanisms and Clinical Management.* London: Academic Press, 1989; 97–115.
25. Bienkowski MJ, Petro MA, Robinson LJ. Inhibition of thromboxane A synthesis in U937 cells by glucocorticoids. *J Biol Chem* 1989; **264**: 6536–6544.
26. Solito E, Raugei G, Melli M, Parente L. Dexamethasone induces the expression of the mRNA of lipocortin 1 and 2 and the release of lipocortin 1 and 5 in differentiated, but not undifferentiated U-937 cells. *FEBS Lett* 1991; **291**: 238–244.
27. Adcock IM, Brown CR, Gelder CM, Shirasaki H, Peters MJ, Barnes PJ. Effects of glucocorticoids on transcription factor activation in human blood mononuclear cells. *Am J Physiol* 1995; **268**: C331–C338.
28. Adcock IM, Shirasaki H, Gelder CM, Peters MJ, Brown CR, Barnes PJ. The effects of glucocorticoids on phorbol ester and cytokine stimulated transcription factor activation in human lung. *Life Sci* 1994; **55**: 1147–1153.
29. Goppelt-Struebe M, Wolter D, Resch K. Glucocorticoids inhibit prostaglandin synthesis not only at the level of phospholipase $A_2$ but also at the level of cyclo-oxygenase/PGE isomerase. *Br J Pharmacol* 1989; **98**: 1287–1295.
30. Hidi R, Vargaftig B, Touqui L. Increased synthesis and secretion of a 14-kDa phospholipase $A_2$ by guinea pig alveolar macrophages. *J Immunol* 1993; **151**: 5613–5623.

**Received 15 August 1997;
accepted in revised form 28 August 1997**
